# Subcellular Trafficking of the Papillomavirus Genome during Initial Infection: The Remarkable Abilities of Minor Capsid Protein L2

**DOI:** 10.3390/v9120370

**Published:** 2017-12-03

**Authors:** Samuel K. Campos

**Affiliations:** 1 The Department of Immunobiology, The University of Arizona, Tucson, AZ 85721-0240, USA; skcampos@email.arizona.edu; Tel.: +1-520-626-4842; 2 The Department of Molecular & Cellular Biology, The University of Arizona, Tucson, AZ 85721-0240, USA; 3 The Cancer Biology Graduate Interdisciplinary Program, The University of Arizona, Tucson, AZ 85721-0240, USA; 4 The BIO5 Institute, Tucson, AZ 85721-0240, USA

**Keywords:** human papillomavirus, HPV16, L2, subcellular trafficking, mitosis, transmembrane domain, translocation, membrane penetration, toxin, fusion peptide, gamma secretase, retromer

## Abstract

Since 2012, our understanding of human papillomavirus (HPV) subcellular trafficking has undergone a drastic paradigm shift. Work from multiple laboratories has revealed that HPV has evolved a unique means to deliver its viral genome (vDNA) to the cell nucleus, relying on myriad host cell proteins and processes. The major breakthrough finding from these recent endeavors has been the realization of L2-dependent utilization of cellular sorting factors for the retrograde transport of vDNA away from degradative endo/lysosomal compartments to the Golgi, prior to mitosis-dependent nuclear accumulation of L2/vDNA. An overview of current models of HPV entry, subcellular trafficking, and the role of L2 during initial infection is provided below, highlighting unresolved questions and gaps in knowledge.

## 1. Introduction

HPVs infect and replicate in cutaneous and mucosal epithelium (skin and oral/genital mucosa). Of the hundreds of HPV types [1,2,3], a set of about 15 HPV “high-risk” types are associated with cervical, anogenital, and oropharyngeal cancers. An additional set of “low-risk” mucosal types cause benign anogenital warts. HPVs are currently the most common sexually transmitted infection, and collectively, these viruses account for 5% of cancers worldwide [4,5,6].

As for most other DNA viruses, a successful HPV infection requires that the viral genome (vDNA) be transported from an extracellular encapsidated state (i.e., viral particles) to a free unencapsidated state within the host cell nucleus, to allow for viral gene expression and vDNA replication. The non-enveloped HPV capsid, comprised of two proteins (L1 and L2), is the molecular machine that accomplishes this task. Seventy-two pentamers of the major capsid protein L1 form the 55 nm icosahedral particle, which together with L2, encapsidate the vDNA. The minor capsid protein L2 is present in variable, but low, amounts, with a maximal occupancy of 72 molecules per virion [7]. Most studies report a range between 12 and 60 molecules of L2 per virion [7,8], and my lab generally estimates 20–40 copies of L2 per particle as measured by Coomassie staining of SDS-PAGE gels and densitometry. Although CryoEM reconstructions indicate that the bulk of L2 density resides beneath the capsid surface underneath the L1 pentamers [7], it is important to remember that regions of L2 are known to be exposed on the surface of the virion [8,9,10]. Likewise, it is important to consider that individual L2 molecules can likely assume different conformations or configurations within the virion, although this has yet to be proven.

**Figure 1:**
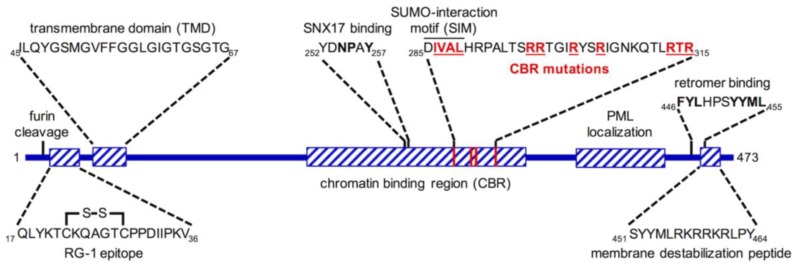
Diagram of the L2 protein. Positions of key components are illustrated. Relative distances and positions are to scale. Chromatin binding mutations are bolded and underlined in red to highlight the residues that were substituted.

L2 is thought to be physically complexed to the vDNA within viral particles, and is responsible for the intracellular transport and nuclear accumulation of the vDNA during infection [11]. Although many studies have reported in vitro DNA-binding activity for the conserved, positively charged N- and C-termini of L2 [12,13,14,15], the structural nature of the L2/vDNA complex within the actual virion remains poorly understood. This review focuses on the remarkable actions of the L2 protein (Figure 1) and the molecular mechanisms and cellular pathways of subcellular trafficking of the L2/vDNA subviral complex. Recent progress will be summarized and outstanding questions and inconsistencies will be highlighted.

It should be noted that many of the findings summarized in this review were found using differentiation-independent pseudovirus (PsV) or quasivirus (QsV) systems for generating infectious or reporter-containing HPV virions in 293TT cells [16]. While more “native” systems for the generation of HPV virions like organotypic raft systems might be ideal, the 293TT PsV/QsV system has been invaluable for basic research on HPV, as the system enables high titer production of highly purified infectious particles, as well as the means to generate virions that package L2 fusions, non-infectious L2 mutants, and 5-ethynyl-2′-deoxyuridine (EdU)-labeled vDNA for a variety of experimental systems. For a recent review on the differences between PsV/QsV and raft-derived HPV, see [17].

## 2. Viral Entry

**Figure 2:**
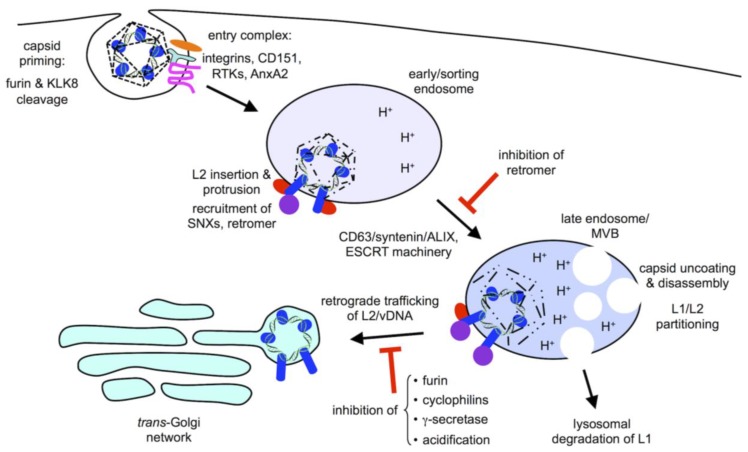
Early subcellular trafficking and uncoating. Internalized virions, primed by cleavage on the cell surface, enter the endolysosomal pathway and begin pH-dependent uncoating and L2 insertion/penetration. L2 recruitment of cytosolic sorting factors including sorting nexins (SNXs) and retromer modulates the trafficking pathway. Retromer binding is important for EE to LE/MVB transport. Retrograde transport of L2/vDNA from LE/MVBs to the *trans*-Golgi network (TGN) occurs in a furin-, cyclophilin- γ-sec-, and pH-dependent manner.

Virion binding to extracellular heparin sulfate proteoglycans (HSPGs) induces conformational changes in both the L1 and L2 capsid proteins of the viral particle, and subsequent transfer of the virion to a cell surface entry receptor complex [18,19,20]. While bound, cell surface kallekrein-8 (KLK8) and furin cleave the L1 and L2 capsids, respectively, an important “priming” event that ensures the proper subsequent subcellular trafficking of L2/vDNA [21,22,23]. Endocytosis of the virion occurs through an actin-dependent process with similarities to macropinocytosis [24]. Tetraspanin CD151 and its associated α_3_β_1_, α_6_β_1_ and α_6_β_4_ integrin partners, growth factor receptor tyrosine kinases, annexin A2, and the cytoskeletal adaptor obscurin-like 1 (OBSL1) have been implicated in tetraspanin-enriched microdomain (TEM)-dependent HPV16 entry [25,26,27,28,29,30,31,32] (Figure 2). While endocytosis of individual cell surface-bound particles can be quite rapid, overall bulk population-level internalization is asynchronous and slow, occurring on the time scale of many hours [24]. CD151 and the associated TEMs likely coordinate organization and assembly of entry receptor complexes; once assembled and bound to virion, these complexes facilitate rapid entry. Similar scaffolding roles for other tetraspanins have been reported for other enveloped and nonenveloped viruses [33,34].

Cleavage of L2 by the host protease furin occurs on the surface of host cells and on the extracellular matrix (ECM) in response to binding of the virion to HSPGs [35]. This cleavage occurs C-terminal to the final arginine residue of a conserved consensus site (RTKR, residues 9–12 for HPV16), removing twelve N-terminal residues of HPV16 L2 [36] (Figure 1). The molecular basis for the requirement of this cleavage remains unknown, but inhibition of cleavage through mutation of the cleavage site or by biochemical inhibition of furin results in aberrant trafficking of the L2/vDNA complex and potent abrogation of infection [23]. Cleavage appears to trigger a conformational change in capsid and/or L2 structure, as the conserved and neutralizing RG-1 epitope (residues 17–36 for HPV16 [37]) becomes accessible to antibody staining shortly after virion binding in a furin-dependent manner [38]. Cell surface cyclophilins (peptidyl-prolyl isomerases, PPIs) also appear to modulate the conformation of L2, as RG-1 epitope exposure is sensitive to cyclosporine A, a broad PPI inhibitor [39]. RG-1 epitope exposure was initially believed to be a convenient marker for furin cleavage, and cyclophilins were believed to control L2 accessibility and susceptibility to furin, but recent work disfavors this idea, as furin cleavage still occurs despite PPI inhibition of RG-1 exposure [36]. Thus, while RG-1 staining is a convenient marker for an L2 conformational change that is both furin- and cyclophilin-dependent, it is not a direct readout for furin proteolysis of L2, as cleavage can occur without RG-1 exposure.

## 3. Subcellular Trafficking

Shortly after entry, early trafficking of HPV is modulated by the tetraspanin CD63 and its partners syntenin/ALIX [40]. These molecules are necessary for sorting of HPV virions from early endosomes (EE) into acidic late endosome (LE) and multivesicular bodies (MVBs), a prerequisite for capsid disassembly, uncoating, and segregation of L2/vDNA from L1 (Figure 2). When MVB trafficking of HPV was interrupted by knockdown of either CD63 or syntenin, subcellular transport of L2/vDNA was altered and infection was partially blocked, demonstrating a requirement for virion transport into MVBs [40]. Accordingly, components of the ESCRT machinery, a group of cytosolic multisubunit complexes that facilitate endosomal maturation and MVB biogenesis, are involved in efficient HPV infection [41,42]. Endosomal acidification is a strict requirement for HPV infection [24,43,44], but it remains unclear if it is simply needed for proper endosomal maturation and MVB biogenesis, or if acidification itself also triggers conformational changes in the HPV capsid or host proteins that are required for downstream processes like capsid uncoating and vDNA trafficking. 

Acid-dependent cathepsin proteases further cleave and process the L1 capsid within the endolysosomal compartments. Capsid proteolysis and disassembly can be visualized by immunofluorescence with the monoclonal antibody L1–7 [45], specific for L1 residues 303–313, located in central cavities underneath each of the L1 pentamers. This region is only available for binding to L1–7 after capsid disassembly; around 8 h post infection [28]. While useful for marking capsid disassembly, L1–7 reactivity does not reveal true infectious uncoating, as staining is blocked by cathepsin inhibitors with no effect on infectivity [22]. Within LE/MVBs the L2/vDNA complex segregates away from the partially degraded L1 capsid in a cyclophilin-dependent manner [46]. The complex then traffics to the trans-Golgi network (TGN) in a retromer-dependent manner [23,47,48], where it resides until the onset of mitosis [49,50,51]. Recent work has shown that a fraction of the L1 capsid, in the form of conformationally intact pentamers, accompanies the L2/vDNA complex to the TGN and nucleus, but a functional role for these L1 pentamers remains unclear [52].

Colocalization of L2/vDNA with TGN markers is well established, but several groups have reported transport of vDNA to more distal retrograde sites. Partial colocalization of vDNA with *cis/medial*-Golgi markers like GM130 and giantin has been reported [23,53]. Likewise, sensitive techniques like the proximity ligation assay [54] have suggested that L2/vDNA retrograde traffics past the Golgi to the ER [55]. Whether these represent primary or alternative routes of infection, or even unproductive dead ends, is not clear. It is worth noting that the dynamic flux of proteins within the secretory compartments makes it difficult to precisely determine where colocalization is occurring by microscopy. Many ER proteins contain a C-terminal KDEL sequence, and although they are maintained within the ER at steady state, they are constantly trafficking into the Golgi, where they must be recycled back through KDEL cargo receptor [56,57]. Sensitive techniques like the proximity ligation assay using such KDEL-containing ER proteins must therefore be interpreted with caution. Since retrograde trafficking of L2/vDNA to more distal compartments has not been well established, this review will simply refer to the final retrograde destination of L2/vDNA as the “TGN”.

## 4. Retromer and Sorting Factors

The retromer, a trimer of Vps26, Vps29, and Vps35, is a cytosolic sorting adaptor complex that binds to peptide motifs within the intracellular domains and cytosolic tails of membrane-bound receptors destined for the TGN. Retromer works in concert with molecules like Rab7b, Rab9a, and members of the sorting nexin family, including SNX3, SNX27, and the BAR-domain sorting nexins (SNX-BAR), to sort cargos from a variety of endosomal compartments to the TGN [58,59]. L2 contains conserved hydrophobic retromer-binding sites near the C-terminus (FYL at residues 446–448 and YYML at residues 452–455 for HPV16, see Figure 1). Mutation of these sites prevents association of L2 with retromer, and blocks the trafficking of L2/vDNA to the TGN, instead causing an accumulation within EEA1-positive endocytic compartments [48] suggesting a retromer-dependent sorting event away from EE compartments (Figure 2). Likewise, siRNA knockdown of retromer components also prevents L2/vDNA from reaching the TGN [48]. In addition to retromer, L2 is capable of interaction with SNX17 and SNX27 to direct endosomal and retrograde trafficking of the vDNA [60,61]. The interaction with SNX17 through a conserved motif (NPxY, residues 254–257 for HPV16, see Figure 1) is believed to occur very early after entry. One recent study observed recruitment of SNX17 to HPV positive endosomes by 2 h post infection, a phenotype that was dependent on the conserved NPxY motif within L2 [62]. The SNX17-L2 interaction likely promotes retention/recycling of the L2/vDNA complex within the endosomal compartment, preventing the rapid trafficking and degradation of L2/vDNA within lysosomal compartments [60]. Perhaps the virion requires a relatively long retention time in moderately acidic EE and LE/MVB environments for efficient uncoating, partitioning of the L2/vDNA subviral complex from L1, and/or recruitment of the retromer? Mutation of the NPxY motif or knockdown of SNX17 results in aberrant L2/vDNA trafficking and decreased infectivity [60].

SNX17 uses its FERM domain to bind to cargo harboring the NPxY motif. SNX27 is another FERM domain-containing SNX involved in L2/vDNA trafficking but, unlike SNX17, SNX27 does not interact with L2 through the conserved NPxY motif. In addition to a FERM domain, SNX27 also contains a PDZ domain, which mediates interaction with L2 through a non-canonical PDZ ligand located somewhere in between residues 192–292 of HPV16 L2 [61]. Notably, both SNX17 and SNX27 have been implicated in efficient retrograde trafficking through the retromer [59,63], raising the possibility that cooperative interactions of L2 with these SNXs may somehow promote retromer-dependent TGN localization.

Recent work has revealed the existence of an additional trimeric sorting complex called the retriever, which functions in concert with SNX17, the CCC, and WASH complexes to sort cargo from degradative to recycling compartments. Retriever consists of three subunits-DSCR3, C16orf62, and Vps29, a subunit in common with the retromer [64]. HPV infection is decreased upon knockdown of retriever components DSCR3 and C16orf62, as well as CCC components CCDC 22 and CCDC93, suggesting a role for this novel pathway in HPV infection [64].

## 5. γ-Secretase

ESCRT proteins, tetraspanins, SNXs, and retromer all have physiological roles in subcellular trafficking and protein transport, and thus many of these natural pathways and components are exploited and commandeered by different viruses for entry or assembly. Perhaps the most mysterious host factor necessary for HPV infection is the multisubunit intramembrane protease γ-secretase (γ-sec), which appears to be a unique requirement of papillomaviruses. The γ-sec complex is a transmembrane protease comprised of four subunits: presenilin1/2 (PS1/2), nicastrin (Nic), Aph1a/b, and PEN2. Two isoforms exist for both PS and Aph1, so there is heterogeneity among cellular γ-sec complexes [65]. γ-sec catalyzes the intramembrane cleavage of TMDs from a wide variety of membrane proteins [66], and is perhaps best known as an important component of the Notch signaling pathway and the biogenesis of Aβ peptides from amyloid precursor protein (APP) [67,68,69,70]. Biochemical inhibition of γ-sec or knockdown of any of the four subunits results in a potent block of HPV infection [55,71]. In a screen of a diverse panel of 34 different mucosal and cutaneous HPV types, sensitivity to γ-sec inhibition was the most conserved feature among the 29 alpha and 5 beta HPV types tested, even higher than sensitivity to furin inhibition [72]. The molecular basis for the γ-sec requirement is unknown, but inhibition of γ-sec activity results in a failure of L2/vDNA to reach the TGN, even though L2 appears to exit EEA1 positive endosomal compartments [55] (Figure 2). This is in contrast to retromer knockdown, which causes an accumulation of vDNA within EEA1 endosomes [48]. This may suggest that, in the absence of γ-sec activity, L2/vDNA never exits the MVB/LE compartments, and instead continues to lysosomes for degradation; or that γ-sec controls trafficking of L2/vDNA to a discrete intermediate compartment, between the MVB and the TGN. Consistent with the observed effects on L2/vDNA trafficking, HPV16 is only sensitive to γ-sec inhibition during the first 6–8 h of infection [55], and γ-sec inhibition has no effect on post-TGN trafficking of vDNA [51]. Failure of L2/vDNA to reach the TGN in the absence of γ-sec activity means that the retrograde trafficking pathway utilized by HPV involves more than just the canonical players like retromer and SNXs, suggesting that new γ-sec-dependent retrograde pathways may exist and are being exploited by papillomaviruses. The catalytic PS1/2 subunit of γ-sec are known to modulate protein trafficking, lysosomal maturation, and Ca^2+^ homeostasis independently of γ-sec-activity, but direct connections to retrograde trafficking pathways are scant [73]. Retromer has, however, been implicated in retrograde-dependent trafficking and cleavage of gamma secretase substrates, including APP [74,75].

## 6. L2 Is an “Inducible Transmembrane” Protein

How does L2, the minor capsid protein from a non-enveloped virus, complexed with the vDNA within the lumen of intracellular vesicular compartments, interact with a variety of cytosolic sorting molecules to direct its own transport to the TGN? Evidence from multiple laboratories suggests that L2 can interact with and span across vesicular membranes, thereby allowing L2 to gain access to the cytosol, to recruit cytosolic factors necessary for retrograde trafficking (Figure 2). Post-TGN transit, full translocation across the limiting membrane, and nuclear accumulation of the L2/vDNA complex requires mitosis and is summarized in greater detail in latter sections of this review. For the sake of consistency within the field, the following nomenclature is proposed for the 3-step process describing L2’s remarkable ability to shift from being a soluble protein to a transmembrane protein and back again:

**(1)**  **Insertion**—Within the lumen, part(s) of L2 insert(s) into the local membrane.
**(2)**  **Protrusion**—L2 becomes a transmembrane protein. Part of L2 remains lumenal and is complexed with the vDNA, while other parts of L2 stick through the membrane and are accessible to cytosolic proteins.
**(3)**  **Translocation**—L2/vDNA exits vesicular compartments, passing across the limiting membrane to establish infection within the cell nucleus.

## 7. Insertion and Protrusion of L2

How does the L2 protein initially interact with and insert into membranes? When naturally or ectopically expressed, L2 is a soluble nuclear protein, not a membrane protein [76,77]. Yet, during infection, L2 must somehow interact with and cross membranes; how is this accomplished? In 2006, a conserved “membrane destabilizing peptide” near the C-terminus of L2 (SYYMLRKRRKRLPY, residues 451–464 for HPV16, see Figure 1) was identified as having a role in the endosomal escape of L2/vDNA via membrane disruption or destabilization [78]. Using synthetic C-terminal peptides from HPV33 L2, containing the corresponding membrane destabilization moiety (SYFILRRRRKRFPYFFTDVRVAA, residues 445–467), in vitro cytotoxicity and propidium iodide uptake experiments showed a pH-dependent ability to disrupt cellular membranes. While it is possible that this C-terminal region aids in membrane insertion of L2 within the acidic endosomes, it is important to note that a direct role in L2 membrane insertion has yet to be demonstrated.

Regardless of how L2 initially inserts into membranes during infection, multiple groups have published indirect evidence that L2 interacts with cytosolic factors and thus must protrude into the cytosol across vesicular membranes. In 2013, my laboratory identified a glycine-rich transmembrane-like domain (TMD) towards the N-terminus of L2 (ILQYGSMGVFFGGLGIGTGSGTG, residues 45–67 of HPV16, see Figure 1) [79]. Taking advantage of TMD-flanking monoclonal antibody epitopes and using elegant immunofluorescence staining procedures, the Sapp laboratory has since demonstrated that L2 utilizes this TMD to span intracellular vesicular membranes with residues C-terminal of the TMD being cytosolic, consistent with a type-I transmembrane topology [80]. Moreover, additional data from the Sapp laboratory suggest the vDNA remains lumenal within these intracellular vesicles [81]. Exactly how L2 is able to insert into the membrane and span across remains unknown, but endosomal acidification seems to be required to adopt this conformation [80], although it remains to be determined if this may simply reflect a requirement for L1 capsid disassembly or L1/L2 partitioning, rather than low pH having a direct effect on L2 protein structure or conformation. Thus, virion-associated L2 appears to be an inducible transmembrane protein, with the ability to insert into membranes and adopt a transmembrane configuration, to drive vDNA subcellular trafficking by physically linking the lumenal vDNA to host cytosolic sorting proteins (Figure 2).

L2’s function to facilitate vDNA delivery across the limiting membrane is analogous to that of many bacterial toxins, which penetrate intracellular membranes and deliver toxin domains to the cytosol. Many of these bacterial toxins including diphtheria toxin, anthrax toxin protective antigen, Shiga toxin, and *Pseudomonas* exotoxin A [82,83] also rely on proteolytic activation by furin and other proteases to trigger conformational changes and structural rearrangements that underlie toxin membrane insertion and penetration. Given the requirement for furin in TGN localization of L2/vDNA, it is very likely that cleavage triggers a structural change that enables L2 to insert and protrude into the local membrane via the TMD to recruit cytosolic SNXs and retromer. Until structural data on L2 is obtained, the nature of any cleavage-induced conformational changes will remain elusive.

Although direct evidence is lacking, the TMD itself may play a role in the initial insertion of L2 into membranes. It is noteworthy that the L2 TMD is quite similar to the fusion peptides (FPs) from many type-I fusogenic glycoproteins of enveloped viruses of the *Orthomyxoviridae*, *Paramyxoviridae*, and *Retroviridae* families [79]. These fusion peptides generally consist of ~20 apolar residues, and are typically enriched for glycine, a composition believed to impart conformational flexibility. The structurally dynamic nature of these fusion peptides is thought to be critical for their ability to partition into and destabilize local membranes [84,85,86,87]. It is also noteworthy that, like L2, these viral fusion proteins require “priming” by proteolytic cleavage and “activation” by environmental cues like low pH or receptor binding [88]. It is conceptually challenging to envision how L2 could achieve a type-I transmembrane state upon insertion of its N-terminal TMD—L2 would have to essentially drag 400 residues C-terminal to the TMD across the membrane. Perhaps cooperative interactions between the TMD, the C-terminal membrane disruption peptide, and the membrane are required for insertion and protrusion of L2.

How does γ-sec facilitate L2/vDNA trafficking? No interaction between L2 and the γ-sec complex has been reported, although it is tempting to envision that L2 could interact with the complex via its TMD while protruding through the membrane. Alternatively, γ-sec activity could somehow be required for L2 to initially insert into membranes to achieve membrane protrusion. This latter possibility would be consistent with the trafficking defects observed upon inhibition of gamma secretase [48], as failure to insert and protrude through the membrane would be expected to block TGN localization, and may instead cause trafficking away from EEA1 positive early endosomes into a degradative lysosomal pathway. Although no evidence exists supporting a role for γ-sec in HPV endocytosis, it has been found as part of a “tetraspanin interactome”, associated with many of the same molecules believed to be part of the initial entry receptor complex, including tetraspanins CD9 and CD81, integrins α3 and β1, and annexin-A2 [89]. The γ-sec complex may therefore be present locally during uncoating when L2 presumably adopts the protruding conformation (Figure 2). In this scenario, one could also imagine L2 actually being a substrate for γ-sec cleavage, triggering a conformational change of some kind upon cleavage of the L2 TMD. While attractive, there is no published evidence supporting this notion. This begs the question- if L2 is not a substrate for γ-sec then how does inhibition of γ-sec catalysis affect L2/vDNA trafficking so drastically? Perhaps γ-sec cleavage of a cellular protein somehow modulates trafficking, or L2 may actually be a “pseudosubstrate”, interacting with γ-sec without cleavage. TMD substrates of γ-sec are believed to first dock into a substrate binding site prior to transfer to the catalytic active site for proteolysis [90]. γ-sec inhibitors perturb the global structure of the γ-sec complex and may therefore prevent initial substrate docking [91,92].

As discussed above, many host proteins and pathways are expoited by HPV to facilitate virion trafficking and viral infection, but some proteins can restrict HPV infection suggesting an inherent anti-papillomaviral function. The endosomal protein stannin restricts HPV infection by rerouting virions away from the TGN to degradative compartments [93]. Similarly, the α-defensin HD5 alters L2/vDNA trafficking, accelerating the degradation of virions within LE/lysosomal compartments to restrict HPV infection [94]. Stannin appears to work by blocking association of L2 with retromer, to prevent retrograde trafficking of L2/vDNA, causing an increased accumulation of L2/vDNA in LAMP1-positive lysosomal compartments [93]. This is in contrast to retromer knockdown or mutation of retromer binding sites in L2, which instead cause a trafficking block within EEA1-positive endosomal compartments [48]. Rather than directly inhibiting the L2-retromer association, stannin likely blocks the insertion and protrusion of L2 within vesicular membranes to indirectly prevent binding and recruitment of retromer. Similarly, HD5 may induce aberrant trafficking of L2/vDNA by directly binding the virion to interfere with L2 insertion and protrusion [94]. Interestingly, interferon gamma has recently been found to restrict HPV infection in an L2-dependent and type-specific manner, decreasing the proteolytic degradation of L1 capsid and causing a block of L2/vDNA in LE/MVB/lysosomal compartments [95]. Cathepsin proteases also appear to limit HPV, as infection is increased upon genetic knockout, siRNA silencing, or biochemical inhibition [22,96]; this is contrary to other non-enveloped viruses, like reoviruses and adeno-associated viruses, which depend on these endosomal proteases for uncoating [97,98]. Vimentin is another recently reported inhibitory factor, found to limit infection at the level of viral entry [99]. Much will be gained from further mechanistic studies of these inhibitory host factors.

## 8. Post-TGN Transport- Mitosis, Translocation, and Chromatin Binding

Prior to 2013, the consensus view was that the L2/vDNA complex egressed from endosomal compartments into the cytosol, as is the case for many other non-enveloped viruses [100,101,102]. Thus, discovery of the TGN as an important stop in the retrograde route of incoming L2/vDNA was a major advance in the field [23,47]. Initially, the L2/vDNA was believed to penetrate the TGN directly and wait in the cytosol prior to nuclear entry. Cell cycle progression into mitosis is known to be important for HPV infection [103], and nuclear envelope breakdown was thought to facilitate L2/vDNA transfer from the cytosol into the nucleus like some retroviruses [50,104].

**Figure 3:**
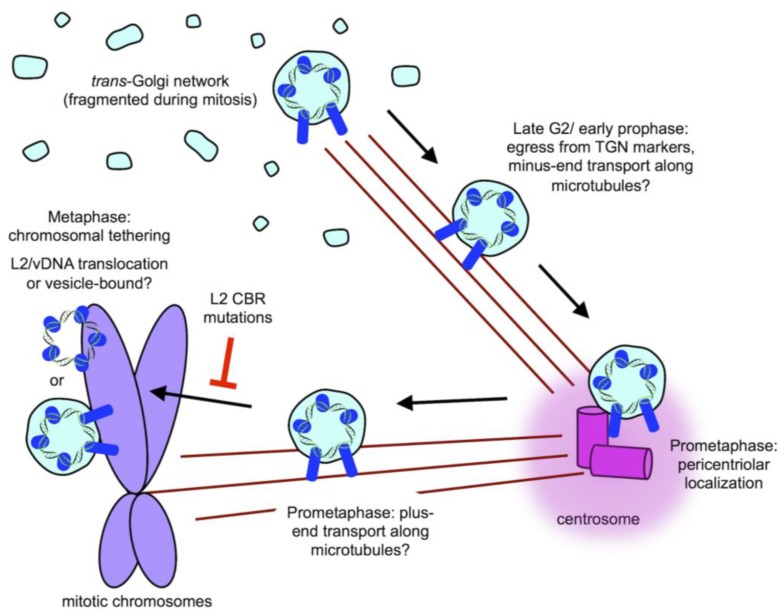
Post-TGN mitotic trafficking of L2/vDNA complex. Upon entry into mitosis, L2/vDNA remains vesicle-bound but loses coincidence with TGN markers. These L2/vDNA-containing vesicles likely travel along astral microtubules in the minus-end direction towards the centrosome, where they accumulate during prometaphase. The vesicles likely switch polarity and travel along the spindle microtubules in the plus-end direction to reach the host chromosomes by metaphase. Chromosome-bound L2/vDNA partitions with host chromosomes, eventually localizing to PML bodies of the daughter cells. Chromosome binding of L2/vDNA is through the chromatin binding region (CBR) of L2 and mutation of this region causes a block in translocation, with vesicular L2/vDNA becoming reabsorbed back into the nascent Golgi after mitosis. Chromosome-bound L2/vDNA may be in a membrane-bound vesiclular state, or may have penetrated the limiting membrane upon chromosome binding, further work is needed to clarify this stage of the HPV life cycle.

Recent work supports a model where, at the onset of mitosis, when the Golgi and TGN naturally begins to fragment and vesiculate, the vesicle-bound vDNA egresses from what was the interphase TGN (Figure 3). The Sapp laboratory pioneered an EdU/vDNA staining technique based on selective membrane permeabilization and sequential EdU labeling to demonstrate the lumenal state of the vDNA [81]. While the exact nature of these vesicles remains unknown, immunofluorescence microscopy reveals that these vDNA-containing vesicles stain negative for classical TGN markers like TGN46 and p230, and appear to migrate along microtubules, clustering around the centrioles during progression from G2/M to prometaphase [51,81]. L2 likely remains in the protruding conformation, spanning across the limiting membrane to coordinate microtubule-dependent traffic of these vDNA-containing vesicles along the mitotic spindle [81]. These vesicles eventually make their way to the condensed chromosomes, and by metaphase, vDNA can be seen to be associated with and presumably bound to the host chromosomes [49,51,81] (Figure 3). From there, the chromosome-bound vDNA is partitioned into daughter cells. In this manner, infection of both daughter cells is favored at an MOI > 2.

When does the L2/vDNA complex fully translocate across the limiting membrane? While useful for observing the trafficking of vDNA during HPV infection, standard subcellular localization of EdU-labeled vDNA by microscopy is insufficient to reveal the actual translocation event. Using their specialized immunofluorescence protocol for sequential fluorophore-azide conjugation of EdU-labeled vDNA in differentially permeabilized cells, the Sapp laboratory concluded that vDNA became cytosolic sometime during G1, well after mitosis [81]. In this model, L2/vDNA would reside within these unique mitotic transport vesicles, bound to chromosomes for an extended period of time (Figure 3), until translocation occurred sometime in G1 after completion of mitosis.

To better understand translocation, my laboratory has developed an alternative platform to detect and measure this elusive process. Our system is based on the biotin-protein ligase BirA, from *Escherichia coli*. The BirA enzyme will covalently attach a biotin molecule to a specific lysine residue of a short peptide substrate, termed the biotin acceptor peptide (BAP). The BAP is a specific substrate for bacterial BirA, and is not recognized by mammalian biotin-protein ligases [51,105]. By generating HPV pseudoviruses that encapsidate a functional L2-BirA fusion and a HaCaT keratinocyte line that stably expresses a cytosolic GFP-BAP fusion, we have set up a two-component compartmentalization assay to detect L2-BirA translocation. In this system, lumenal L2-BirA is separated from the cytosolic GFP-BAP substrate by limiting membranes. Only upon translocation of L2 will BirA encounter the BAP; thus, biotinylation of GFP-BAP is a readout for translocation. However, it should be noted that, since the assay relies on the fusion of BirA to the extreme C-terminus of L2, biotinylation of GFP-BAP could result from cytosolic exposure of just the C-terminus of L2, rather than full translocation of the entire L2/vDNA complex.

Using this system, we found that L2 translocation required TGN localization of L2/vDNA and cell cycle progression past G2/M. Timecourse experiments with synchronized HaCaT-GFP-BAP cells demonstrated that the earliest biotinylated GFP-BAP signal was detected at or just prior to the onset of mitosis. Although these experiments were performed with a bulk population, we believe it suggests that L2 translocation (or at least of the C-terminus of L2) begins during mitosis (Figure 3), well before transition into G1. Moreover, this timing of L2/vDNA translocation would be consistent with the visual “jump” of vDNA from a punctate pericentriolar distribution in prometaphase to being chromosome-bound by metaphase [51].

The chromosome binding ability of L2 was first reported in 2014 [50], and since then, the Schelhaas group has mapped a minimal chromatin binding region (CBR, residues 188–334 for HPV16) within L2 [49]. Interestingly, the ability of ectopically expressed L2-GFP fusion to associate with mitotic chromatin was found to require cell cycle progression into prometaphase. This finding is suggestive that either the interaction between L2 and mitotic chromatin is indirect, requiring a prometaphase-specific factor, or that L2 is post-translationally modified during prometaphase to somehow activate its chromatin binding ability. Substitution of specific residues (IVAL; 286–289, R302/305, and RTR; 313–315) were found to completely abrogate the chromatin binding activity while mutation of RR396/397 resulted in a partial-inhibition CBR function. When these same residues were mutated in reporter-expressing PsV, packaged with either L2 or L2-BirA, the same phenotypes were observed—infectivity and translocation were completely blocked for IVAL; 286–289, R302/305, and RTR; 313–315 and partially blocked for RR396/397 [49,51].

The striking correlation between the ability of L2 to bind chromatin and to translocate during infection supports a model whereby chromatin binding is required for L2 translocation [49,51]. A mechanistic linkage between these processes favors a model where translocation of L2/vDNA out of the mitotic vesicles occurs while these compartments encounter mitotic chromosomes during prometaphase, and is supported by appearance of translocation signal in timecourse experiments [51]. While further work is needed to validate one model over another, it should be noted that the two models do not have to be mutually exclusive. Translocation studies based on L2-BirA may in fact only be revealing exposure of the L2 C-terminus, and full translocation of vDNA could be occurring at a later time post-mitosis, as suggested by the Sapp laboratory. Alternatively, the nature of these vesicles is unknown, and if their lipid composition and detergent solubility changes during mitosis, it could affect sequential labeling efficiency or EdU-labeled vDNA availability to fluorophore-azides after differential detergent permeabilization.

## 9. A Topology Conundrum

**Figure 4:**
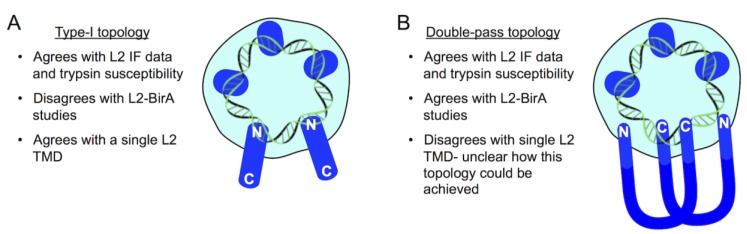
Topology models of L2 protrusion. Both models are consistent with published L2 immunofluorescence and trypsin susceptibility data [80]. (**A**) In the type-I model, the N-terminus remains lumenal with all ~400 residues downstream of the TMD being cytosolic to recruit sorting factors. L2-BirA would be expected to biotinylate substrate in this model, contradicting the actual data [51]. (**B**) In the double-pass model, both the N- and C-termini would be lumenal, with the bulk of L2 being cytosolic. L2-BirA would not be expected to biotinylate substrate as observed. However, the means by which L2 spans the membrane a second time is difficult to conceptualize, as the protein only has one TMD towards the N-terminus [79]. In vitro data suggest both the N- and C-termini are capable of non-speciific dsDNA binding through electrostatic interactions [12,14].

As mentioned above, immunofluorescence staining with mAbs specific for L2 epitopes flanking the TMD suggest a type-I topology for L2 protrusion, with the N-terminal ~45 residues being lumenal, a ~25 residue TMD, and the C-terminal ~400 residues being cytosolic [80] (Figure 4). This topology is in agreement with the placement of known SNX17 and retromer binding motifs within L2, as well as the newly defined CBR (Figure 1). However, translocation studies with PsV encapsidating the L2-BirA fusion are suggestive of a different topology for L2. HPV infection in the presence of S-phase blockers like aphidicolin traps incoming L2/vDNA at the TGN, likely in the protruding conformation [51]. This block is reversible, as removal of the drug releases cell cycle inhibition and enables synchronized egress and translocation of L2/vDNA out of the TGN upon entry into mitosis [51]. This data, however, is not in agreement with a strict type-I membrane topology for L2. The lack of translocation signal in the presence of aphidicolin implies that the C-terminus of L2-BirA is not cytosolic, as it would be in a type-I topology. Rather, it suggests that BirA is either lumenal or is somehow obstructed from engaging the GFP-BAP substrate when L2 is protruding from endosomal and TGN compartments, only becoming accessible to the cytosol upon entry into mitosis. A double-pass topology of protruding L2-BirA would result in a lumenal C-terminal BirA (Figure 4).

It should be noted that the C-terminal membrane destabilization peptide bears no resemblance to a conventional TMD, and no other membrane-spanning regions of L2 have been identified, so it is unclear how L2 could span the membrane twice to keep the C-terminal BirA fusion lumenal. Membrane-spanning bacterial toxins, including anthrax toxin, Diphtheria toxin, botulinum toxin, tetanus toxin, and *Clostridium difficile* toxins, form pores through which they can extrude themselves into the cytosol by a variety of protein translocation mechanisms [106,107]. Oligomerization of individual L2 molecules, each with a single membrane-spanning TMD, could theoretically enable a double-pass topology by extrusion of the L2 C-termini back into the lumen through such a pore. This hypothetical configuration would place a central portion of L2 within the cytosol to recruit sorting factors and direct traffic of the associated vDNA (Figure 4). It should also be noted that such a double-pass topology would still be consistent with the immunofluorescence data supporting a type-I topology [80]. Clearly, much more work is needed to understand the protruding conformation of L2 during HPV infection.

## 10. PML Bodies and Beyond

Regardless of the precise mechanisms of L2 translocation, the minor capsid protein eventually leaves vesicular compartments and is seen along with vDNA within interphase nuclei of infected cells, localized to punctate nuclear foci called promyelocytic leukemia (PML) nuclear domains, also known as PML oncogenic domains (PODS), or ND10 bodies [108]. PML bodies are small nuclear structures, organized by the PML protein for which they are named. These dynamic domains are present in most cells, and are assembled and remodeled in response to a variety of cellular stresses, including infection, innate immune triggers/interferon (IFN), heat shock, DNA damage pathways, and metabolic stress [109,110,111]. PML bodies modulate a wide variety of cellular responses via recruitment, retention, and modification of numerous proteins including the transcriptional repressor Daxx, tumor suppressor Sp100, transcriptional regulator ATRX, DNA helicase BLM, kinase HIPK2, and a multitude of other host proteins. The PML protein, which has many different isoforms, is critical to the assembly of PML bodies and recruitment of host proteins to these foci [112]. Many PML-associated proteins are either directly conjugated to small ubiquitin-like modifier (SUMO) proteins or contain short linear SUMO-interaction motifs (SIMs), or both. In addition to PML oligomerization, SUMOylation and SUMO-SIM networks are believed to be important to PML assembly and dynamics [113].

Given the role of PML bodies in innate antiviral responses, many viruses have been shown to target PML bodies or induce degradation or remodeling of specific PML components [114,115]. PML bodies have been shown to be important for efficient infection from reporter-expressing HPV16 pseudoviruses, as well as authentic BPV virions [108], suggesting that the vDNA is actively targeted to these sites by L2 upon infection. Ectopically expressed L2 can localize to PML bodies, remodeling them through recruitment of Daxx and depletion of Sp100 [116]. In older studies, the ability of GFP-L2 fusions to localize and induce remodeling of PML bodies was mapped to a C-terminal region of L2 (residues 360–420 for HPV33) [117]. L2 can itself be SUMOylated at a conserved lysine residue (K35 for HPV16) when ectopically expressed, but recent work suggests this modification is not important for PML body localization [118,119]. Rather, a moderately conserved SIM (DIVAL, residues 285–289 for HPV16, see Figure 1) has been implicated in PML localization of ectopically expressed untagged full length, L2 [118]. Precise mechanisms of L2-dependent PML body remodeling have yet to be worked out but ectopic overexpression studies must be interpreted with caution since the mode of L2 gene transfection/delivery has been shown to heavily influence nuclear/PML localization of L2 [120]. Recent work suggests that the PML component Sp100 restricts HPV transcription and vDNA replication [121], favoring a model whereby incoming L2 might promote a nuclear environment conducive for early HPV gene transcription and genome maintenance in basal cells.

During cell division, PML bodies show increased dynamics and disperse into the cytosol during open mitosis. Only after exit from mitosis and reformation of the nuclear envelope are PML bodies assembled de novo, and recruitment of Daxx and Sp100 is observed [122]. Whether L2 recruits PML to nucleate de novo assembly of PML bodies in the vicinity of the vDNA after mitosis or whether the L2/vDNA complex is targeted to newly formed PML bodies in early G1 remains to be determined. Likewise, much remains to be discovered regarding preferential remodeling of PML components like Daxx and Sp100 immediately after mitotic translocation of L2/vDNA, and the consequences of this remodeling for infection, immune evasion, and viral persistence.

## 11. Conclusions & Future Directions

In addition to role(s) in vDNA packaging, virion assembly, and particle stability [11], minor capsid protein L2 is tasked with ensuring nuclear delivery of the vDNA during HPV infection. This feat is accomplished via some remarkable means for a viral capsid protein present in low copy numbers. L2 is able to partition vDNA away from degradative endolysosomal compartments, instead diverting it to the TGN. L2 does this by possessing properties of an “inducible transmembrane” protein, with the ability to insert into and protrude across local vesicular membranes using a transmembrane-like domain. Portions of L2 containing conserved sorting motifs are exposed to the cytosol, recruiting cellular sorting factors that dictate retrograde trafficking of L2/vDNA to the TGN. Upon entry into mitosis, the vesicular L2/vDNA complex separates from the dispersed Golgi towards the pericentriolar region and by metaphase the vDNA can be seen to be associated with condensed chromosomes. Whether the visual association of vDNA with mitotic chromosomes represents full translocation of L2/vDNA across limiting membranes, or simply vDNA-filled post-Golgi vesicles bound to chromosomes, remains to be shown. Together with recent work on the chromatin-binding abilities of L2, translocation studies using a novel BirA-based approach suggest that chromatin binding is necessary for translocation. Timecourse experiments with synchronized cells suggest that translocation is concurrent with or slightly after the onset of mitosis. In contrast, sequential fluor-azide conjugation of EdU-labeled vDNA after differential detergent permeabilization suggests that translocation, as defined by the liberation of vDNA from membrane-bound compartments, occurs post-mitosis in G1. Regardless of the specific mechanisms and timing of translocation, L2/vDNA localizes to PML bodies of the daughter cells and likely functions to promote efficient viral gene expression.

Additional efforts are needed to further define the mechanisms of L2’s remarkable abilities. Structural studies are needed to understand the nature of the L2/vDNA complex within viral particles, the molecular basis underlying the requirement for furin, the consequences of cleavage, and the mechanisms of L2-membrane interaction. Further work is needed to understand and define the nature of L2 protrusion through limiting membranes, specifically the topology of L2 within these membranes, and to identity any host proteins that may be necessary for L2-membrane insertion and protrusion. More work is necessary to identify the nature of the post-Golgi vesicles in which L2 resides upon entry into mitosis, and again to define the host proteins that may be interacting with L2 to enable subcellular transport of these compartments and eventual translocation. Elucidation of the timing and mechanisms of actual L2/vDNA translocation will require new approaches. Finally, the precise role(s) of virion-derived L2 in the remodeling of PML bodies, establishment of infection, initial viral gene expression, and potential immunoevasion all represent exciting avenues of future endeavors.

## References

[1] Bzhalava D., Muhr L.S., Lagheden C., Ekstrom J., Forslund O., Dillner J., Hultin E. (2014). Deep sequencing extends the diversity of human papillomaviruses in human skin. Sci. Rep..

[2] Van Doorslaer K. (2013). Evolution of the papillomaviridae. Virology.

[3] Van Doorslaer K., Li Z., Xirasagar S., Maes P., Kaminsky D., Liou D., Sun Q., Kaur R., Huyen Y., McBride A.A. (2017). The papillomavirus episteme: A major update to the papillomavirus sequence database. Nucleic Acids Res..

[4] Doorbar J., Quint W., Banks L., Bravo I.G., Stoler M., Broker T.R., Stanley M.A. (2012). The biology and life-cycle of human papillomaviruses. Vaccine.

[5] Forman D., de Martel C., Lacey C.J., Soerjomataram I., Lortet-Tieulent J., Bruni L., Vignat J., Ferlay J., Bray F., Plummer M. (2012). Global burden of human papillomavirus and related diseases. Vaccine.

[6] Schiffman M., Doorbar J., Wentzensen N., de Sanjose S., Fakhry C., Monk B.J., Stanley M.A., Franceschi S. (2016). Carcinogenic human papillomavirus infection. Nat. Rev. Dis. Prim..

[7] Buck C.B., Cheng N., Thompson C.D., Lowy D.R., Steven A.C., Schiller J.T., Trus B.L. (2008). Arrangement of L2 within the papillomavirus capsid. J. Virol..

[8] Bywaters S.M., Brendle S.A., Tossi K.P., Biryukov J., Meyers C., Christensen N.D. (2017). Antibody competition reveals surface location of HPV L2 minor capsid protein residues 17–36. Viruses.

[9] Pastrana D.V., Gambhira R., Buck C.B., Pang Y.Y., Thompson C.D., Culp T.D., Christensen N.D., Lowy D.R., Schiller J.T., Roden R.B. (2005). Cross-neutralization of cutaneous and mucosal papillomavirus types with anti-sera to the amino terminus of L2. Virology.

[10] Kondo K., Ishii Y., Ochi H., Matsumoto T., Yoshikawa H., Kanda T. (2007). Neutralization of HPV16, 18, 31, and 58 pseudovirions with antisera induced by immunizing rabbits with synthetic peptides representing segments of the HPV16 minor capsid protein L2 surface region. Virology.

[11] Wang J.W., Roden R.B. (2013). L2, the minor capsid protein of papillomavirus. Virology.

[12] Bordeaux J., Forte S., Harding E., Darshan M.S., Klucevsek K., Moroianu J. (2006). The L2 minor capsid protein of low-risk human papillomavirus type 11 interacts with host nuclear import receptors and viral DNA. J. Virol..

[13] Fay A., Yutzy W.H., Roden R.B.S., Moroianu J. (2004). The positively charged termini of L2 minor capsid protein required for bovine papillomavirus infection function separately in nuclear import and DNA binding. J. Virol..

[14] Klucevsek K., Daley J., Darshan M.S., Bordeaux J., Moroianu J. (2006). Nuclear import strategies of high-risk HPV18 L2 minor capsid protein. Virology.

[15] Zhou J., Sun X.Y., Louis K., Frazer I.H. (1994). Interaction of human papillomavirus (HPV) type 16 capsid proteins with HPV DNA requires an intact L2 N-terminal sequence. J. Virol..

[16] Buck C.B., Thompson C.D. (2007). Production of papillomavirus-based gene transfer vectors. Curr. Protoc. Cell Biol..

[17] Biryukov J., Meyers C. (2015). Papillomavirus infectious pathways: A comparison of systems. Viruses.

[18] Aksoy P., Gottschalk E.Y., Meneses P.I. (2017). HPV entry into cells. Mutat. Res. Rev. Mutat. Res..

[19] Day P.M., Schelhaas M. (2014). Concepts of papillomavirus entry into host cells. Curr. Opin. Virol..

[20] DiGiuseppe S., Bienkowska-Haba M., Guion L.G., Sapp M. (2017). Cruising the cellular highways: How human papillomavirus travels from the surface to the nucleus. Virus Res..

[21] Richards R.M., Lowy D.R., Schiller J.T., Day P.M. (2006). Cleavage of the papillomavirus minor capsid protein, L2, at a furin consensus site is necessary for infection. Proc. Natl. Acad. Sci. USA.

[22] Cerqueira C., Samperio Ventayol P., Vogeley C., Schelhaas M. (2015). Kallikrein-8 proteolytically processes human papillomaviruses in the extracellular space to facilitate entry into host cells. J. Virol..

[23] Day P.M., Thompson C.D., Schowalter R.M., Lowy D.R., Schiller J.T. (2013). Identification of a role for the trans-Golgi network in human papillomavirus 16 pseudovirus infection. J. Virol..

[24] Schelhaas M., Shah B., Holzer M., Blattmann P., Kuhling L., Day P.M., Schiller J.T., Helenius A. (2012). Entry of human papillomavirus type 16 by actin-dependent, clathrin- and lipid raft-independent endocytosis. PLoS Pathog..

[25] Aksoy P., Abban C.Y., Kiyashka E., Qiang W., Meneses P.I. (2014). HPV16 infection of HaCaTs is dependent on β4 integrin, and α6 integrin processing. Virology.

[26] Dziduszko A., Ozbun M.A. (2013). Annexin A2 and S100A10 regulate human papillomavirus type 16 entry and intracellular trafficking in human keratinocytes. J. Virol..

[27] Scheffer K.D., Gawlitza A., Spoden G.A., Zhang X.A., Lambert C., Berditchevski F., Florin L. (2013). Tetraspanin CD151 mediates papillomavirus type 16 endocytosis. J. Virol..

[28] Spoden G., Freitag K., Husmann M., Boller K., Sapp M., Lambert C., Florin L. (2008). Clathrin- and caveolin-independent entry of human papillomavirus type 16—Involvement of tetraspanin-enriched microdomains (tems). PLoS ONE.

[29] Spoden G., Kuhling L., Cordes N., Frenzel B., Sapp M., Boller K., Florin L., Schelhaas M. (2013). Human papillomavirus types 16, 18, and 31 share similar endocytic requirements for entry. J. Virol..

[30] Surviladze Z., Dziduszko A., Ozbun M.A. (2012). Essential roles for soluble virion-associated heparan sulfonated proteoglycans and growth factors in human papillomavirus infections. PLoS Pathog..

[31] Woodham A.W., da Silva D.M., Skeate J.G., Raff A.B., Ambroso M.R., Brand H.E., Isas J.M., Langen R., Kast W.M. (2012). The S100A10 subunit of the annexin A2 heterotetramer facilitates L2-mediated human papillomavirus infection. PLoS ONE.

[32] Wustenhagen E., Hampe L., Boukhallouk F., Schneider M.A., Spoden G.A., Negwer I., Koynov K., Kast W.M., Florin L. (2016). The cytoskeletal adaptor obscurin-like 1 interacts with the human papillomavirus 16 (HPV16) capsid protein L2 and is required for HPV16 endocytosis. J. Virol..

[33] Earnest J.T., Hantak M.P., Li K., McCray P.B., Perlman S., Gallagher T. (2017). The tetraspanin CD9 facilitates mers-coronavirus entry by scaffolding host cell receptors and proteases. PLoS Pathog..

[34] Feneant L., Levy S., Cocquerel L. (2014). CD81 and hepatitis c virus (HCV) infection. Viruses.

[35] Day P.M., Schiller J.T. (2009). The role of furin in papillomavirus infection. Future Microbiol..

[36] Bronnimann M.P., Calton C.M., Chiquette S.F., Li S., Lu M., Chapman J.A., Bratton K.N., Schlegel A.M., Campos S.K. (2016). Furin cleavage of L2 during papillomavirus infection: Minimal dependence on cyclophilins. J. Virol..

[37] Gambhira R., Karanam B., Jagu S., Roberts J.N., Buck C.B., Bossis I., Alphs H.H., Culp T., Christensen N.D., Roden R.B.S. (2007). A protective and broadly cross-neutralizing epitope of human papillomavirus L2. J. Virol..

[38] Day P.M., Gambhira R., Roden R.B., Lowy D.R., Schiller J.T. (2008). Mechanisms of human papillomavirus type 16 neutralization by L2 cross-neutralizing and L1 type-specific antibodies. J. Virol..

[39] Bienkowska-Haba M., Patel H.D., Sapp M. (2009). Target cell cyclophilins facilitate human papillomavirus type 16 infection. PLoS Pathog..

[40] Gräßel L., Fast L.A., Scheffer K.D., Boukhallouk F., Spoden G.A., Tenzer S., Boller K., Bago R., Rajesh S., Overduin M. (2016). The CD63-syntenin-1 complex controls post-endocytic trafficking of oncogenic human papillomaviruses. Sci. Rep..

[41] Broniarczyk J., Bergant M., Gozdzicka-Jozefiak A., Banks L. (2014). Human papillomavirus infection requires the TSG101 component of the ESCRT machinery. Virology.

[42] Broniarczyk J., Pim D., Massimi P., Bergant M., Gozdzicka-Jozefiak A., Crump C., Banks L. (2017). The VPS4 component of the ESCRT machinery plays an essential role in hpv infectious entry and capsid disassembly. Sci. Rep..

[43] Muller K.H., Spoden G.A., Scheffer K.D., Brunnhofer R., de Brabander J.K., Maier M.E., Florin L., Muller C.P. (2014). Inhibition by cellular vacuolar ATPase impairs human papillomavirus uncoating and infection. Antimicrob. Agents Chemother..

[44] Smith J.L., Campos S.K., Wandinger-Ness A., Ozbun M.A. (2008). Caveolin-1-dependent infectious entry of human papillomavirus type 31 in human keratinocytes proceeds to the endosomal pathway for pH-dependent uncoating. J. Virol..

[45] Sapp M., Kraus U., Volpers C., Snijders P.J., Walboomers J.M., Streeck R.E. (1994). Analysis of type-restricted and cross-reactive epitopes on virus-like particles of human papillomavirus type 33 and in infected tissues using monoclonal antibodies to the major capsid protein. J. Gen. Virol..

[46] Bienkowska-Haba M., Williams C., Kim S.M., Garcea R.L., Sapp M. (2012). Cyclophilins facilitate dissociation of the human papillomavirus type 16 capsid protein L1 from the L2/DNA complex following virus entry. J. Virol..

[47] Lipovsky A., Popa A., Pimienta G., Wyler M., Bhan A., Kuruvilla L., Guie M.A., Poffenberger A.C., Nelson C.D., Atwood W.J. (2013). Genome-wide siRNA screen identifies the retromer as a cellular entry factor for human papillomavirus. Proc. Natl. Acad. Sci. USA.

[48] Popa A., Zhang W., Harrison M.S., Goodner K., Kazakov T., Goodwin E.C., Lipovsky A., Burd C.G., DiMaio D. (2015). Direct binding of retromer to human papillomavirus type 16 minor capsid protein L2 mediates endosome exit during viral infection. PLoS Pathog..

[49] Aydin I., Villalonga-Planells R., Greune L., Bronnimann M.P., Calton C.M., Becker M., Lai K.Y., Campos S.K., Schmidt M.A., Schelhaas M. (2017). A central region in the minor capsid protein of papillomaviruses facilitates viral genome tethering and membrane penetration for mitotic nuclear entry. PLoS Pathog..

[50] Aydin I., Weber S., Snijder B., Samperio Ventayol P., Kuhbacher A., Becker M., Day P.M., Schiller J.T., Kann M., Pelkmans L. (2014). Large scale RNAi reveals the requirement of nuclear envelope breakdown for nuclear import of human papillomaviruses. PLoS Pathog..

[51] Calton C.M., Bronnimann M.P., Manson A.R., Li S., Chapman J.A., Suarez-Berumen M., Williamson T.R., Molugu S.K., Bernal R.A., Campos S.K. (2017). Translocation of the papillomavirus L2/vDNA complex across the limiting membrane requires the onset of mitosis. PLoS Pathog..

[52] DiGiuseppe S., Bienkowska-Haba M., Guion L.G.M., Keiffer T.R., Sapp M. (2017). Human papillomavirus major capsid protein L1 remains associated with the incoming viral genome throughout the entry process. J. Virol..

[53] Ishii Y., Nakahara T., Kataoka M., Kusumoto-Matsuo R., Mori S., Takeuchi T., Kukimoto I. (2013). Identification of TrappC8 as a host factor required for human papillomavirus cell entry. PLoS ONE.

[54] Lipovsky A., Zhang W., Iwasaki A., DiMaio D. (2015). Application of the proximity-dependent assay and fluorescence imaging approaches to study viral entry pathways. Methods Mol. Biol..

[55] Zhang W., Kazakov T., Popa A., DiMaio D. (2014). Vesicular trafficking of incoming human papillomavirus 16 to the Golgi apparatus and endoplasmic reticulum requires γ-secretase activity. mBio.

[56] Gomez-Navarro N., Miller E. (2016). Protein sorting at the ER-Golgi interface. J. Cell Biol..

[57] Villeneuve J., Duran J., Scarpa M., Bassaganyas L., van Galen J., Malhotra V. (2017). Golgi enzymes do not cycle through the endoplasmic reticulum during protein secretion or mitosis. Mol. Biol. Cell.

[58] Bonifacino J.S., Hurley J.H. (2008). Retromer. Curr. Opin. Cell Biol..

[59] Burd C., Cullen P.J. (2014). Retromer: A master conductor of endosome sorting. Cold Spring Harb. Perspect. Biol..

[60] Bergant Marusic M., Ozbun M.A., Campos S.K., Myers M.P., Banks L. (2012). Human papillomavirus L2 facilitates viral escape from late endosomes via sorting nexin 17. Traffic.

[61] Pim D., Broniarczyk J., Bergant M., Playford M.P., Banks L. (2015). A novel PDZ domain interaction mediates the binding between human papillomavirus 16 L2 and sorting nexin 27 and modulates virion trafficking. J. Virol..

[62] Bergant M., Peternel S., Pim D., Broniarczyk J., Banks L. (2017). Characterizing the spatio-temporal role of sorting nexin 17 in human papillomavirus trafficking. J. Gen. Virol..

[63] Yin W., Liu D., Liu N., Xu L., Li S., Lin S., Shu X., Pei D. (2012). SNX17 regulates notch pathway and pancreas development through the retromer-dependent recycling of jag1. Cell Regen..

[64] McNally K.E., Faulkner R., Steinberg F., Gallon M., Ghai R., Pim D., Langton P., Pearson N., Danson C.M., Nagele H. (2017). Retriever is a multiprotein complex for retromer-independent endosomal cargo recycling. Nat. Cell Biol..

[65] De Strooper B., Iwatsubo T., Wolfe M.S. (2012). Presenilins and γ-secretase: Structure, function, and role in alzheimer disease. Cold Spring Harb. Perspect. Med..

[66] Beel A.J., Sanders C.R. (2008). Substrate specificity of γ-secretase and other intramembrane proteases. Cell. Mol. Life Sci..

[67] Andrew R.J., Kellett K.A., Thinakaran G., Hooper N.M. (2016). A greek tragedy: The growing complexity of alzheimer amyloid precursor protein proteolysis. J. Biol. Chem..

[68] De Strooper B., Annaert W., Cupers P., Saftig P., Craessaerts K., Mumm J.S., Schroeter E.H., Schrijvers V., Wolfe M.S., Ray W.J. (1999). A presenilin-1-dependent gamma-secretase-like protease mediates release of notch intracellular domain. Nature.

[69] Herreman A., Serneels L., Annaert W., Collen D., Schoonjans L., De Strooper B. (2000). Total inactivation of gamma-secretase activity in presenilin-deficient embryonic stem cells. Nat. Cell Biol..

[70] Zhang Z., Nadeau P., Song W., Donoviel D., Yuan M., Bernstein A., Yankner B.A. (2000). Presenilins are required for γ-secretase cleavage of β-APP and transmembrane cleavage of Notch-1. Nat. Cell Biol..

[71] Karanam B., Peng S., Li T., Buck C., Day P.M., Roden R.B. (2010). Papillomavirus infection requires gamma secretase. J. Virol..

[72] Kwak K., Jiang R., Wang J.W., Jagu S., Kirnbauer R., Roden R.B. (2014). Impact of inhibitors and L2 antibodies upon the infectivity of diverse alpha and beta human papillomavirus types. PLoS ONE.

[73] Duggan S.P., McCarthy J.V. (2016). Beyond γ-secretase activity: The multifunctional nature of presenilins in cell signalling pathways. Cell. Signal..

[74] Choy R.W., Cheng Z., Schekman R. (2012). Amyloid precursor protein (APP) traffics from the cell surface via endosomes for amyloid β (Aβ) production in the trans-Golgi network. Proc. Natl. Acad. Sci. USA.

[75] Small S.A., Gandy S. (2006). Sorting through the cell biology of alzheimer’s disease: Intracellular pathways to pathogenesis. Neuron.

[76] Auvinen E., Kujari H., Arstila P., Hukkanen V. (1992). Expression of the L2 and E7 genes of the human papillomavirus type 16 in female genital dysplasias. Am. J. Pathol..

[77] Hagensee M.E., Yaegashi N., Galloway D.A. (1993). Self-assembly of human papillomavirus type 1 capsids by expression of the L1 protein alone or by coexpression of the L1 and L2 capsid proteins. J. Virol..

[78] Kämper N., Day P.M., Nowak T., Selinka H.C., Florin L., Bolscher J., Hilbig L., Schiller J.T., Sapp M. (2006). A membrane-destabilizing peptide in capsid protein L2 is required for egress of papillomavirus genomes from endosomes. J. Virol..

[79] Bronnimann M.P., Chapman J.A., Park C.K., Campos S.K. (2013). A transmembrane domain and GxxxG motifs within L2 are essential for papillomavirus infection. J. Virol..

[80] DiGiuseppe S., Keiffer T.R., Bienkowska-Haba M., Luszczek W., Guion L.G., Muller M., Sapp M. (2015). Topography of the human papillomavirus minor capsid protein L2 during vesicular trafficking of infectious entry. J. Virol..

[81] DiGiuseppe S., Luszczek W., Keiffer T.R., Bienkowska-Haba M., Guion L.G., Sapp M.J. (2016). Incoming human papillomavirus type 16 genome resides in a vesicular compartment throughout mitosis. Proc. Natl. Acad. Sci. USA.

[82] Garred O., van Deurs B., Sandvig K. (1995). Furin-induced cleavage and activation of shiga toxin. J. Biol. Chem..

[83] Gordon V.M., Klimpel K.R., Arora N., Henderson M.A., Leppla S.H. (1995). Proteolytic activation of bacterial toxins by eukaryotic cells is performed by furin and by additional cellular proteases. Infect. Immun..

[84] Epand R.M. (2003). Fusion peptides and the mechanism of viral fusion. Biochim. Biophys. Acta.

[85] Tamm L.K., Han X., Li Y., Lai A.L. (2002). Structure and function of membrane fusion peptides. Biopolymers.

[86] Lorieau J.L., Louis J.M., Bax A. (2010). The complete influenza hemagglutinin fusion domain adopts a tight helical hairpin arrangement at the lipid:Water interface. Proc. Natl. Acad. Sci. USA.

[87] Hofmann M.W., Weise K., Ollesch J., Agrawal P., Stalz H., Stelzer W., Hulsbergen F., de Groot H., Gerwert K., Reed J. (2004). De novo design of conformationally flexible transmembrane peptides driving membrane fusion. Proc. Natl. Acad. Sci. USA.

[88] White J.M., Delos S.E., Brecher M., Schornberg K. (2008). Structures and mechanisms of viral membrane fusion proteins: Multiple variations on a common theme. Crit. Rev. Biochem. Mol. Biol..

[89] Wakabayashi T., Craessaerts K., Bammens L., Bentahir M., Borgions F., Herdewijn P., Staes A., Timmerman E., Vandekerckhove J., Rubinstein E. (2009). Analysis of the γ-secretase interactome and validation of its association with tetraspanin-enriched microdomains. Nat. Cell Biol..

[90] Wolfe M.S. (2010). Structure, mechanism and inhibition of gamma-secretase and presenilin-like proteases. Biol. Chem..

[91] Kornilova A.Y., Das C., Wolfe M.S. (2003). Differential effects of inhibitors on the γ-secretase complex. Mechanistic implications. J. Biol. Chem..

[92] Li Y., Bohm C., Dodd R., Chen F., Qamar S., Schmitt-Ulms G., Fraser P.E., St George-Hyslop P.H. (2014). Structural biology of presenilin 1 complexes. Mol. Neurodegener..

[93] Lipovsky A., Erden A., Kanaya E., Zhang W., Crite M., Bradfield C., MacMicking J., DiMaio D., Schoggins J.W., Iwasaki A. (2017). The cellular endosomal protein stannin inhibits intracellular trafficking of human papillomavirus during virus entry. J. Gen. Virol..

[94] Wiens M.E., Smith J.G. (2017). α-defensin HD5 inhibits human papillomavirus 16 infection via capsid stabilization and redirection to the lysosome. mBio.

[95] Day P.M., Thompson C.D., Lowy D.R., Schiller J.T. (2017). Interferon gamma prevents infectious entry of human papillomavirus 16 via an L2-dependent mechanism. J. Virol..

[96] Calton C.M., Schlegel A.M., Chapman J.A., Campos S.K. (2013). Human papillomavirus type 16 does not require cathepsin L or B for infection. J. Gen. Virol..

[97] Akache B., Grimm D., Shen X., Fuess S., Yant S.R., Glazer D.S., Park J., Kay M.A. (2007). A two-hybrid screen identifies cathepsins B and L as uncoating factors for adeno-associated virus 2 and 8. Mol. Ther. J. Am. Soc. Gene Ther..

[98] Ebert D.H., Deussing J., Peters C., Dermody T.S. (2002). Cathepsin L and cathepsin B mediate reovirus disassembly in murine fibroblast cells. J. Biol. Chem..

[99] Schafer G., Graham L.M., Lang D.M., Blumenthal M.J., Bergant Marusic M., Katz A.A. (2017). Vimentin modulates infectious internalization of human papillomavirus 16 pseudovirions. J. Virol..

[100] Schiller J.T., Day P.M., Kines R.C. (2010). Current understanding of the mechanism of HPV infection. Gynecol. Oncol..

[101] Smith A.E., Helenius A. (2004). How viruses enter animal cells. Science.

[102] Tsai B. (2007). Penetration of nonenveloped viruses into the cytoplasm. Annu. Rev. Cell Dev. Biol..

[103] Pyeon D., Pearce S.M., Lank S.M., Ahlquist P., Lambert P.F. (2009). Establishment of human papillomavirus infection requires cell cycle progression. PLoS Pathog..

[104] Lewis P.F., Emerman M. (1994). Passage through mitosis is required for oncoretroviruses but not for the human immunodeficiency virus. J. Virol..

[105] Schatz P.J. (1993). Use of peptide libraries to map the substrate specificity of a peptide-modifying enzyme: A 13 residue consensus peptide specifies biotinylation in *Escherichia coli*. Nat. Biotechnol..

[106] Murphy J.R. (2011). Mechanism of diphtheria toxin catalytic domain delivery to the eukaryotic cell cytosol and the cellular factors that directly participate in the process. Toxins.

[107] Pirazzini M., Azarnia Tehran D., Leka O., Zanetti G., Rossetto O., Montecucco C. (2016). On the translocation of botulinum and tetanus neurotoxins across the membrane of acidic intracellular compartments. Biochim. Biophys. Acta.

[108] Day P.M., Baker C.C., Lowy D.R., Schiller J.T. (2004). Establishment of papillomavirus infection is enhanced by promyelocytic leukemia protein (PML) expression. Proc. Natl. Acad. Sci. USA.

[109] Lallemand-Breitenbach V., de The H. (2010). PML nuclear bodies. Cold Spring Harb. Perspect. Biol..

[110] Sahin U., Lallemand-Breitenbach V., de The H. (2014). PML nuclear bodies: Regulation, function and therapeutic perspectives. J. Pathol..

[111] Scherer M., Stamminger T. (2016). Emerging role of PML nuclear bodies in innate immune signaling. J. Virol..

[112] Weidtkamp-Peters S., Lenser T., Negorev D., Gerstner N., Hofmann T.G., Schwanitz G., Hoischen C., Maul G., Dittrich P., Hemmerich P. (2008). Dynamics of component exchange at PML nuclear bodies. J. Cell Sci..

[113] Sahin U., Ferhi O., Jeanne M., Benhenda S., Berthier C., Jollivet F., Niwa-Kawakita M., Faklaris O., Setterblad N., de The H. (2014). Oxidative stress-induced assembly of pml nuclear bodies controls sumoylation of partner proteins. J. Cell Biol..

[114] Everett R.D. (2001). DNA viruses and viral proteins that interact with PML nuclear bodies. Oncogene.

[115] Everett R.D., Chelbi-Alix M.K. (2007). PML and PML nuclear bodies: Implications in antiviral defence. Biochimie.

[116] Florin L., Schafer F., Sotlar K., Streeck R.E., Sapp M. (2002). Reorganization of nuclear domain 10 induced by papillomavirus capsid protein L2. Virology.

[117] Becker K.A., Florin L., Sapp C., Sapp M. (2003). Dissection of human papillomavirus type 33 L2 domains involved in nuclear domains (ND) 10 homing and reorganization. Virology.

[118] Bund T., Spoden G.A., Koynov K., Hellmann N., Boukhallouk F., Arnold P., Hinderberger D., Florin L. (2014). An L2 sumo interacting motif is important for PML localization and infection of human papillomavirus type 16. Cell. Microbiol..

[119] Marusic M.B., Mencin N., Licen M., Banks L., Grm H.S. (2010). Modification of human papillomavirus minor capsid protein L2 by sumoylation. J. Virol..

[120] Kieback E., Muller M. (2006). Factors influencing subcellular localization of the human papillomavirus L2 minor structural protein. Virology.

[121] Stepp W.H., Meyers J.M., McBride A.A. (2013). Sp100 provides intrinsic immunity against human papillomavirus infection. mBio.

[122] Chen Y.C., Kappel C., Beaudouin J., Eils R., Spector D.L. (2008). Live cell dynamics of promyelocytic leukemia nuclear bodies upon entry into and exit from mitosis. Mol. Biol. Cell.

